# How Can the EU Beating Cancer Plan Help in Tackling Lung Cancer, Colorectal Cancer, Breast Cancer and Melanoma?

**DOI:** 10.3390/healthcare10091618

**Published:** 2022-08-25

**Authors:** Denis Horgan, Anne-Marie Baird, Mark Middleton, Zhasmina Mihaylova, Jan P. Van Meerbeeck, Jens Vogel-Claussen, Paul E. Van Schil, Josep Malvehy, Paolo Antonio Ascierto, France Dube, Michael Zaiac, Jonathan A. Lal, Grażyna Kamińska-Winciorek, Marco Donia, Thierry André, Marta Kozaric, Pia Osterlund, Dan Lucian Dumitrascu, Luca Bertolaccini

**Affiliations:** 1 European Alliance for Personalised Medicine, 1040 Brussels, Belgium; 2 Department of Molecular and Cellular Engineering, Jacob Institute of Biotechnology and Bioengineering, Faculty of Engineering and Technology, Sam Higginbottom University of Agriculture, Technology and Sciences, Prayagraj 211007, India; 3 Lung Cancer Europe, 3008 Bern, Switzerland; 4 School of Medicine, Trinity Translational Medicine Institute, Trinity College Dublin, Dublin 2, Ireland; 5 Department of Oncology, Cancer Research UK Oxford Centre, University of Oxford, Oxford OX3 7DQ, UK; 6 Department of Medical Oncology, Military Medical Academy, 1000 Sofia, Bulgaria; 7 Department of Thoracic Oncology, Antwerp University Hospital, 2650 Edegem, Belgium; 8 Department of Diagnostic and Interventional Radiology, Hannover Medical School, 30625 Hannover, Germany; 9 Biomedical Research in Endstage and Obstructive Lung Disease Hannover (BREATH), German Center for Lung Research (DZL), Hannover Medical School, 30625 Hannover, Germany; 10 Department of Thoracic and Vascular Surgery, Antwerp University Hospital, Antwerp University, 2650 Antwerp, Belgium; 11 Melanoma Unit, Dermatology Department Hospital Clinic, University of Barcelona, 08036 Barcelona, Spain; 12 IDIBAPS, CIBER de Enfermedades Raras, Instituto Carlos III, 28029 Madrid, Spain; 13 Unit of Melanoma, Cancer Immunotherapy and Development Therapeutics, Istituto Nazionale Tumori IRCCS Fondazione “G. Pascale”, 80131 Naples, Italy; 14 Astra Zeneca, 1800 Concord Pike, Wilmington, DE 19803, USA; 15 Novartis Farma SpA, 21040 Origgio, Italy; 16 Institute for Public Health Genomics, Department of Genetics and Cell Biology, GROW School of Oncology and Developmental Biology, Faculty of Health, Medicine and Life Sciences, Maastricht University, 6229 ER Maastricht, The Netherlands; 17 Department of Bone Marrow Transplantation and Hematology-Oncology, Skin Cancer and Melanoma Team, M. Sklodowska-Curie National Research Institute of Oncology, 44-101 Gliwice, Poland; 18 National Center for Cancer Immune Therapy, Department of Oncology, Copenhagen University Hospital, 2730 Herlev, Denmark; 19 Department of Medical Oncology, Sorbonne Université, Hôpital Saint-Antoine, AP-HP, 75012 Paris, France; 20 Department of Oncology, Tampere University Hospital, 33520 Tampere, Finland; 21 Department of Upper GI Cancer, Karolinska University Hospital and Institute, 171 76 Stockholm, Sweden; 22 Semiology Departement, 2nd Medical Clinic, University of Medicine and Pharmacy “Iuliu Hatieganu”, 400347 Cluj-Napoca, Romania; 23 Division of Thoracic Surgery, IEO European Institute of Oncology IRCCS, 20141 Milan, Italy

**Keywords:** cancer, EU Beating Cancer Plan, EBCP, personalised medicine, healthcare, policy framework, treatment, screening, reimbursement, molecular diagnostics, unmet medical need, access, patient, citizens

## Abstract

Cancer is the second leading cause of mortality in EU countries, and the needs to tackle cancer are obvious. New scientific understanding, techniques and methodologies are opening up horizons for significant improvements in diagnosis and care. However, take-up is uneven, research needs and potential outstrip currently available resources, manifestly beneficial practices—such as population-level screening for lung cancer—are still not generalised, and the quality of life of patients and survivors is only beginning to be given attention it merits. This paper, mainly based on a series of multistakeholder expert workshops organised by the European Alliance for Personalised Medicine (EAPM), looks at some of those specifics in the interest of planning a way forward. Part of this exercise also involves taking account of the specific nature of Europe and its constituent countries, where the complexities of planning a way forward are redoubled by the wide variations in national and regional approaches to cancer, local epidemiology and the wide disparities in health systems. Despite all the differences between cancers and national and regional resources and approaches to cancer care, there is a common objective in pursuing broader and more equal access to the best available care for all European citizens.

## 1. Introduction

The needs for tackling cancer are obvious—and Europe’s Beating Cancer Plan (EBCP) can certainly contribute to solutions, with its funding of more than EUR 4bn [1] and pathways to additional financing via EU programs on research and on regional and recovery funding. There is no question of the volume and gravity of the needs. Cancer is the second leading cause of mortality in EU countries after cardiovascular diseases [2]. Every year, around 2.6 million people are diagnosed with the disease, and it kills another 1.2 million people [3]. The overall economic burden of cancer in Europe is estimated at more than EUR 100 billion annually. In addition to putting pressure on individuals, national health and social care systems and state budgets, this disease also affects productivity and economic growth [3,4]. New scientific understanding and new techniques and methodologies are opening up horizons for great improvements in diagnosis and care [5]. However, take-up is uneven, research needs and potential outstrip currently available resources, manifestly beneficial practices—such as population-level screening for lung cancer—are still not generalised, and the quality of life of patients and survivors is only beginning to be given attention it merits. The EU can help the Member States in need of evidence-based policy making to ensure that all EU citizens have equal access to high-quality cancer prevention, screening, diagnostics, treatment, and aftercare. Nevertheless, cancer is not just one disease [6], and different forms of cancer present different challenges, which should be taken into account in any policy discussion. Over and above the general actions that can help in the overall combat against cancer, there are particular needs within specific types of cancer [7]. This paper looks at some of those specifics in the interest of planning a way forward. Part of this exercise also involves taking account of the specific nature of Europe and its constituent countries, where the complexities of planning a way forward are redoubled by the wide variations in national and regional approaches to cancer, local epidemiology, and the wide disparities in health systems, on which much improvement depends—including notably the supply-side considerations of resources and expertise for testing, treatment, reimbursement, or infrastructure and the demand-side considerations of incidence, take-up and awareness. This conundrum is the rationale for this paper, based largely on a series of multistakeholder expert workshops organised by the European Alliance for Personalised Medicine (EAPM) earlier in 2022 and examining the possibilities for mobilisation of common efforts to identify gaps and promote improvements across the cancer field, with particular attention to lung cancer, breast cancer, colorectal cancer and malignant melanoma (MM). The time is right to develop cooperation via the EBCP, Horizon Europe, and other EU policy instruments, in synergy with the Member States, regions, and cities, and with foundations, civil society and industry. This could ensure maximum benefit from the available resources, in terms of EU funding from Horizon Europe for Research and Development (R&D) actions, deployment through other Multiannual Financial Framework (MFF) instruments, national/regional financial support, and the de-risking of private investments. In commercial settings, we often see companies that are more operationally efficient acquiring other companies of at least similar or bigger size to improve their operating ratio and hence improve the company performance. This exchange of efficiency, also called bootstrapping, could be one of the major remits of the EBCP. Despite all the differences between cancers and national and regional resources and approaches to cancer care, there is a common objective in pursuing wider and more equal access to the best available care for all European citizens. However, the big challenge that is still present for patients with cancer is the equity of access to screening and therapeutic innovations [8]. Furthermore, much of the mechanisms to achieve this require national as much as—or more than—EU action. Archetypically, even though marketing authorisation in Europe is a centralised process through the European Medicines Agency (EMA), the reimbursement process of innovative therapeutics still occurs at a national level [9]. The job of Europe in much of this is to promote collaboration, demonstrate best practices, encourage improvements and leverage learning out of the recent pandemic. The EBCP aims to ensure that 90% of the eligible EU population are offered screening and, more importantly, that citizens respond to screening for breast, cervical and colorectal cancer by 2025 [10,11]—an admirable objective. Early detection through screening can help save lives, but meeting that goal depends on national action, as screening programmes are set up and run by the member states. Screening has a big role to play in other cancers, too—not least lung and melanoma, as detailed below. However, performance here is a clear example of where the action is needed because inequalities to access persist among the Member States. The number of at-risk people being tested varies among the Member States, ranging (for cervical cancer, for instance) from 25% to 80%, as a function of national priorities, resources and commitment [12]. There are significant inequalities among the population within the countries of the European Union in the areas of early detection, diagnosis, treatment and quality of patient care. A proactive approach is essential to make cancer treatment as successful as possible, with as few side effects as possible, and to ensure long-term survival. However, as detection methods become more sensitive, it can be difficult to distinguish insignificant changes from lesions that will lead to life-threatening cancer. Family history and screening for variants such as *BRCA1*, *BRCA2* and *CDH1*, are currently used to identify individuals at high risk of cancer development. However, recent advances in the epidemiology and genetics of cancer and the routine availability of relevant information through electronic health records mean that for several cancers, it is now possible to use multifactor assessment to provide a more personalized cancer risk for all individuals. After the individual’s risk profile is identified, it can be offered either a specific test for a particular type of cancer or a broader test to look for signals for multiple cancers. Blood sampling provides an alternative approach to proximal sampling directly from or near relevant tissue. An ideal solution would be a single early detection cancer test for multiple cancers, for example, in body fluids such as blood, which could be performed on anyone over a certain age at regular intervals. Such a test should be sensitive enough to detect cancer at an early stage and specific enough to minimize false positive results and determine the likely site in the body. The main limitation of the current biopsy approach is that the number of genes that recurrently mutate in cancer is very low. Another issue is how to know how many markers are needed to detect cancer at an early stage. The authors estimated that an analysis of 500 cancer-specific markers would be required to achieve a similar level of susceptibility in other solid tumors as was shown in the detection of Epstein-Barr Virus DNA in Nasopharyngeal Carcinoma. Some of the other innovative technologies that can be used for early cancer detection are nano tools, new optical modalities for medical imaging, ultrasound imaging for affordable cancer diagnosis, optical imaging and photoacoustic tomography. Future approaches should aim to establish methods for enrichment and easier detection of ctDNA and circulating cancer cells in plasma or urine. By combining more data from clinical data examinations, simple laboratory tests, genomes, proteomes and metabolites screening, intelligent algorithms will be needed to put reasonable numbers into data, find patterns that elude the human eye, and even identify new biomarkers. Since pioneering work on the classification of skin cancer and lung cancer, numerous papers and opinions have explored the benefits and challenges of using artificial intelligence (AI) in the early detection of cancer. It is necessary to ensure that the AI is connected to user-friendly software and to address the incentives and barriers to adopting the AI from patients and clinicians. AI will not eliminate the need for doctors and experts to interpret the findings but will cut the cost and time required to diagnose the disease and will consequently allow doctors to spend more time developing effective and holistic treatment protocols [1,3,13].

At the EU level, major policy initiatives in the health field are underway or in preparation, many of them offering direct or indirect pathways for implementation. Europe’s Beating Cancer Plan (EBCP) offers a policy framework, establishing resource allocation for and thereby improving the implementation of personalized healthcare at the national level. The question is how the EBCP can be best utilised and also what opportunities are provided for different member states to tackle cancer. The aim of the narrative review is to first provide background with general conditions and challenges regarding four cancers (lung, colorectal, breast and melanoma cancers) throughout Europe and then elicit from expert panel members potential steps for implementing the EBCP across Europe.

## 2. Materials and Methods

This paper’s inputs and perspectives are based on a detailed literature search and the multistakeholder expert workshops organised by the European Alliance for Personalised Medicine (EAPM) earlier in 2022. A narrative literature search for articles published between the date of database inception and 31 January 2022 was performed in EMBASE (via Ovid), MEDLINE (via PubMed) and Cochrane CENTRAL. The purpose was to identify specific challenges such as late or incorrect cancer diagnosis, lack of access to appropriate therapies and expertise, lack of commercial feasibility in developing new cancer therapies, difficulties in conducting well-powered clinical studies, availability of tissue banks, etc., in different countries across Europe. Identified records were imported into Mendeley reference management software. The literature search was complemented with the expert panel organised by EAPM, where the experts discussed the literature findings, but the main aim was to address the opportunities that EBCP could provide in tackling these cancers. The EAPM secretariat screened for titles and abstracts of relevant articles on the proposed topics. Full-text copies of potentially eligible reports were separately evaluated by the expert panel members. When multiple studies contained overlapping data, the most informative one was included. Any disagreements were resolved by consensus or arbitration. Other authors listed in the article were experts in their fields who participated in the round table and discussed the presented literature findings.

## 3. Results

Results are grouped into two major categories, describing differences between national health systems and different circumstances for four different cancers (lung, colorectal, breast and melanoma cancer). All the results are based either on the expert discussions from the workshop and/or literature search. Because of that, there are some differences due to the information available from the workshops and the article screening process.

### 3.1. Different National Health Systems

The scale of variations in Europe’s national healthcare systems is precise, as the following brief panorama indicates across the key factors such as organisation of care delivery, availability of products and services, reimbursement and take-up [14].

In Belgium, health care is based on therapeutic freedom for physicians, freedom of choice for patients, and fee-for-service payment. Prevention, screening, teaching and health care organisations are regional competencies, while all disease-oriented care is of federal competence. The well-resourced system is financed through compulsory social insurance covering almost the whole population and administered by a national institute. However, the multilayered, complex health care system and the lack of centralisation of care processes can result in fragmentation of care—although a national electronic health database for physicians feeds institutional medical data onto a single platform. A government commission examines drug pricing applications and advises the Minister of Social Affairs and Public Health, but the Minister of Economic Affairs sets the maximum drug price. To speed up the introduction of innovative medicines where uncertainties persist over their added value, reimbursement decisions increasingly turn to managed entry agreements [15].

In Germany, cancer treatment is provided by several public institutions alongside private oncology practices, which have diagnostic, outpatient treatment and surveillance capacities but not in-patient care [16,17]. Screening is reimbursed for breast, cervical, prostate and colorectal cancers. However, health technology assessment (HTA) of new examination and treatment methods follows strict regulations. For the coverage of medical procedures by German statutory health insurances, a distinction is made between outpatient and in-patient care sectors and outpatient specialist care. Reimbursement by the country’s constituent states is not uniform [18]. According to data from 2013 [19], participation in cancer screening programmes was around 67.2% for women and 40% for men.

The French health care system is financed through compulsory social insurance covering almost the whole population. All medical and administrative information relating to reimbursement is collected in the national health data system [20]. There is no national cancer registry, but a network has been constructed among the distinct cancer registries across the country [21]. After EU authorisation, a new drug must obtain approval from the French Health Authority, where the transparency committee advises on comparative effectiveness. This allows health insurance to set the reimbursement rate and negotiate the price with the pharmaceutical companies [20]. Challenges are expected for national health policy due to the increasing number of cancer immunotherapies. Reimbursement is complicated by uncertainties across different treatments in upcoming new techniques and biomarkers. A temporary use authorisation system provides access to innovative drugs, allowing access for patients with cancer who have no therapeutic option to off-label and as-yet-unavailable drugs [22].

In Italy, the Italian National Health Service (NHS) guarantees universal health care, essentially free at the point of care. The NHS is decentralised and administered through 19 regions and 2 autonomous provinces, which presents challenges for policymakers. Regional governments reimburse hospitals with a lump sum payment for each patient, determined by the patient’s diagnosis, health status and procedures performed [23,24].

Spain’s Ministry of Health and Social Policy is responsible for health policy and enabling legislation. However, the establishment of 17 regional autonomous health services has brought local differences in care delivery. Once a drug is approved by the Spanish Agency of Drugs and Medical Products and a price is agreed upon, each region can decide whether to invest and incorporate the new drug into its regional formulary. In some regions, staff in individual hospitals decide whether to invest in a nationally approved drug. This regional investment in treatment can lead to delays in the availability of new treatment options between regions [25,26].

In Sweden, a national cancer strategy aims at early diagnosis, but the implementation of guidelines by regional administrations means progress has been uneven. There is little priority or promotion of screening for colorectal and lung cancer or other early detection and diagnostic services, and the early part of the care continuum performs poorly. A pharmaceutical benefits board makes reimbursement recommendations for drugs. A national hospital discharge register includes all in-patient care [27,28].

Finland has a not-for-profit healthcare system that delivers most of the care. A national strategy for screening is utilized aiming at early diagnosis of cancer, and for example, a new screening program for colorectal cancer has just been implemented. National guidelines direct cancer diagnosis and treatment and regional variation is modest. Once a drug is approved by the European Agency of Drugs, the Finnish Agency decides whether a national approval is needed, and otherwise, the five-university hospital districts approve the drug and incorporate the new drug into its regional formulary.

Poland has a publicly funded health system, and an HTA agency and an expert advisory committee work together to make drug-funding recommendations to the national Ministry of Health [29].

In 2019, Croatia published a comprehensive national cancer action plan designed to reduce cancer incidence and mortality rates and improve cancer patients’ quality of life [30].

The United Kingdom’s not-for-profit national healthcare system delivers most health care, including cancer diagnosis and treatment, as “free at the point of care”, but through a regional system that creates variations in high-cost health care. The system offers excellent data capture and the ability to map and reveal regional variation, but funding is tied to government expenditure, and infrastructure and workforce investment is urgently needed. Systemic anticancer therapy requires regulatory and health technology appraisal for reimbursement. Private health insurance is uncommon [31,32].

### 3.2. Different Circumstances for Four Different Cancers

Alongside the specific conditions of different national health services and against the general background of cancer care today, this paper focuses on the situation in respect of four of the significant manifestations of cancer: lung, colorectal, breast and melanoma.

#### 3.2.1. Lung Cancer

Lung cancer (LC) is the leading cause of global cancer incidence and mortality worldwide, with an estimated 2.1 million new cases and 1.8 million deaths in 2018 [33]; it is also the leading cause of cancer-related deaths in Europe, responsible for approximately 384,000 deaths in 2020 [34]. Advanced (stage IIIb/IV) non-small-cell lung cancer (NSCLC) has a significant economic impact on society. The total cost from an NSCLC diagnosis to death was estimated at EUR 10,991 (in a range from EUR 7197 for Germany to EUR 27,381 for Sweden) in 2016 Euro values, with drugs accounting for two-thirds of total costs [35]. The introduction of new treatment options, especially over the last 6 years, has likely increased these costs. Risk factors include family history, tobacco smoking (more than three-quarters of LC cases in the Western world are attributable to smoking), and radon from natural underground uranium decay [36]. Lung cancer is the most frequent cancer and the leading cause of cancer death among males, followed by prostate and colorectal cancer (for incidence) and liver and stomach cancer (for mortality). Among females, lung cancer is the third most frequent cancer after breast cancer and colorectal cancer (for incidence and also for mortality) [33]. A stronger understanding of lung cancer epidemiology and risk factors can inform preventive measures and curb the growing disease burden worldwide [37]. Screening of LC is currently mostly oriented at current and ex-smokers, with growing awareness of air pollution as a cause potentially warranting screening in polluted areas. Most European countries have not yet endorsed thoracic computed tomography (CT) screening. However, trials suggest that the potential benefits of systematic early diagnosis of lung cancer through LDCT screening could be around 12.5 years of additional life, even in the presence of comorbidities, with possibly around 22,000 lung cancer deaths prevented in Europe every year [10,11,38,39]. Nevertheless, it needs targeting on groups most likely to benefit and least likely to be harmed, taking into consideration the financial resources available. Revised guidelines in 2021 recommend annual LDCT screening for adults aged 50–80 with a 20 pack-year smoking history—but this depends on the availability of information on risk variables from primary healthcare records or directly from the patient. There is little evidence that Small-Cell Lung Cancer (SCLC) outcomes are improved by CT screening recommendations that reduce overall LC mortality [10,11]. Encouraging data are emerging on the integration of genetic susceptibility pathways and circulating biomarkers in risk-prediction models. However, validated biomarkers are needed along with a move beyond epidemiological and clinical data, requiring not only access to current CT-screening biobanks but also the development of high-quality prospective biobanks embedded in future screening programmes together with radiomics data. Future molecular tests must be validated and cost-effective and potentially use nanotechnology-based approaches [40]. Diagnostic procedures and treatment reimbursement differ among countries, and follow-up can be slow. Nearly half of European LC patients wait more than two months from their first medical consultation to receiving their diagnosis [41]. Treatment options include radiotherapy, surgical resection, chemotherapy and targeted therapy. Surgical tumour resection is often the most effective therapeutic modality in stage I and II NSCLC but is limited in advanced stages and the presence of metastatic spread. Lack of early stage diagnosis means most lung cancers are detected in advanced stages, often with distant metastasis not treatable with surgery. Chemotherapy and radiotherapy have more recently been complemented by targeted molecular therapies, such as tyrosine kinase inhibitors (TKIs) that have reshaped treatment, although resistance has developed in first and second generations [42,43,44]. The early performance with a rapid result turnaround of the next-generation sequencing technique (NGS) into routine laboratory practice would enable a better and wider selection of NSCLC patients for targeted therapies. The continuous development of the molecular markers and their determination in blood-derived, free-circulating tumor DNA (ctDNA) in LC is a suitable model tumor type for druggable mutations and the use of immunotherapies. The detection of druggable mutations has opened new paths to liquid biopsy (LB). Determination of tumour mutational burden has been explored as a predictive biomarker for immunotherapy in NSCLC [45]. Nanotechnology also demonstrates the potential to provide novel and paradigm-shifting solutions to current medical problems. Clinical and biological barriers still need to be overcome, as many alternative interventions’ results have been unsatisfactory. Rapid on-site evaluation can improve diagnostic yield and patient care but requires experienced cytopathology staff [46]. It has been shown that the presence of isolated carcinoma cells detected immuno-cytochemically in the bone marrow is of prognostic relevance for cancer patients. Since the immunocytochemical method (ICC) is laborious and often depends on the subjective interpretation of the individual investigator, an immunoassay was designed for the detection of cytokeratin 19 (CK19). Follow-up studies in breast cancer, non-small-cell lung cancer, esophageal and colorectal cancer, as well as in neuroblastoma, have shown the prognostic significance of isolated carcinoma cells in bone marrow for a clinical relapse [47]. An important factor to be taken into account is a minimal residual disease (MRD), which is a significant problem after a curative tumor re-section. The Union for International Cancer Control (UICC) standardization committee has suggested a new subcategory called Mi (i) which denotes the stage of minimal systemic disease. The prognostic relevance of this new diagnostic approach for MRD has been documented in several clinical studies on lung, breast, colorectal and gastric cancer [48]. 

##### National Variations in LC Experience

Provision for LC testing and treatment varies widely across Europe. In Finland, Germany, Israel, Sweden and the Netherlands, the majority of drugs are approved and reimbursed, but not in eastern Europe, and even Ireland, Portugal, Spain and Italy have five or more drug indications not reimbursed. Access to molecular testing differs, with patients from Croatia, Romania, Poland, Latvia, Turkey and Spain lacking access to three or more molecular tests. There are differences in the number of biomarkers tested and reimbursed for molecular tests in each country, as well as in turnaround times for results. Some countries in Southern and Central-Eastern Europe have minimal access to radiotherapy. Access to a clinical trial depends heavily on the number of trials conducted in a country; patients from Israel, Switzerland, Denmark, Norway and the Netherlands have many more possibilities to access an LC clinical trial than patients from Croatia, Turkey, Germany, Greece and Bulgaria. Anti-smoking legislation is now common in many European countries. Austria, Belgium, Finland, France, Netherlands, Norway, Poland, Romania, Spain, Sweden and UK are performing high or moderately high in the public health domain, which covers awareness, prevention, screening and the role of patient input in policy formation [49,50].

In Poland, LC is rarely diagnosed at the early stages. Late or incorrect diagnosis is aggravated by a lack of access to appropriate therapies and expertise. Recent years have brought new treatment options for patients with non-small-cell LC, such as immunotherapy in stage IV and stage III, but reimbursement is comparatively limited [45,51]. 

In the UK, LC accounts for about 21% of all cancer and continues to be the most common cause of cancer death. LDCT is still not fully funded, and meeting national guidelines recommending a time limit of 28 days from referral to a treatment recommendation is straining the capacity for rapid turnaround of diagnostics. Centrally funded genomic laboratory hubs are replacing regional laboratories for molecular testing, primarily through NGS panels. Surgery research is vital, and there are 61 well-equipped radiotherapy centres. Patient and physician advocacy is also strong, contributing to improved LC outcomes [31]. 

In Croatia, national recommendations provide obligatory predictive testing for all, partly paid for by national health insurance and partly supported by pharmaceutical companies (monoclonal antibodies for immunohisto/cytochemistry) [30,41,52]. 

In Italy, LC currently represents the third most common neoplasm in the overall population, and it is also the first cause of cancer death in men and the third in females [53]. Screening with thoracic CT has not yet been endorsed in Italy but is under discussion. A range of predictive molecular biomarkers are currently approved and reimbursed, but national platforms for molecular screening of patients with NSCLC are not validated. Many molecular pathology laboratories are equipped with a NGS platform, but implementation in the clinical setting is still limited to a small number of large-volume centres. Differences in the regional reimbursement systems affect the implementation of relevant biomarkers analysis in the clinical setting. The lack of standardisation in both test request and reimbursement procedures poses a challenge. In recent years, multidisciplinary tumour boards have begun to appear in some regions [54]. The Italian Network for Lung Cancer Screening has a budget of EUR 2 million in 2021–2022 for funding 18 centres of excellence for lung cancer prevention and monitoring [55]. 

In Belgium, LC screening is organised at the regional level, and there are no official LC screening programs for high-risk (ex)smokers: implementation of population-based screening is needed. The provision of a reimbursement fee has primarily contributed to the successful implementation of LC tumour boards and the creation of integrated multidisciplinary oncology care programs. Access to novel therapies is complex and can be slow. Implementation of patient-reported outcomes and better integration of oncology care pathways are still awaited. Belgium has been slow to develop specialised oncologic surgery, and many smaller hospitals conduct only a few major lung resections a year, despite evidence that high-volume centres perform better. Nevertheless, health authority guidelines have emerged on pharmacodiagnostic molecular testing for different tumor types and its reimbursement, and for LC, an algorithm is proposed with updates every 6 months [15].

In France, LC is the second most common cancer in men and the third in women [56]. The national public health agency publishes a yearly assessment of measures to cut smoking, but there is no official lung screening program. Incomplete coordination among specialists causes delays from detecting a suspicious abnormality to the final diagnosis and tumour staging. All platforms have shifted their routine molecular profiling to NGS, and all private and public laboratories in France can access funding for molecular biology testing in oncology. No oncologic treatment can be delivered without formal MTB written conclusions [20].

LC in Bulgaria is the most common malignancy and the first cause of death from malignancy in men and the second in women [57]. In this country, unfortunately, there are no screening programs for LC, as well as no screening for colorectal, cervical, prostate, breast cancer and melanoma. The biggest problem after diagnosing LC patients is testing the tumor for genetic drivers. There is no state funding for pathoanatomical and genetic laboratories to carry out this activity. Pharmaceutical companies pay different state and private laboratories for PCR and/or ICH testing of various markers, which leads to loss of tumor tissue and interferes with adequate antitumor treatment. For Bulgaria, the marketing authorization date is the date of central European authorization. The time for reimbursement varies from one and more than one year, and the procedure is quite complicated. In 2019, a CECOG survey was conducted in 10 Central and Eastern European (CEE) countries (Austria, Bulgaria, Croatia, Czech Republic, Hungary, Poland, Romania, Serbia, Slovenia and Slovakia), which aimed at investing access to novel anticancer drugs for NSCLC and time from marketing authorization to national reimbursement [58]. The results of the study showed that the time from Medical Assistance (MA) to reimbursement differed between <3 months (only Austria) and >12 months needed for most novel drugs to get reimbursement in the rest nine countries. In addition, according to the Ministry of Finance, public payments for medicines as a percentage (%) of total public payments for health care for the period from 2012 to 2021 do not increase [59]. 

##### LC Needs

Inadequate levels, scope and targeting of LC screening need urgent action, along with the consolidation of standardised data collection systems, clinical cancer registries with outcome data and patient-reported outcome measures (PROMs), improved infrastructure for access to modern radiotherapy, thoracic oncology standardisation and training, the establishment of multidisciplinary teams and quality and peer review of systems, centralisation of programmes for rare tumours or minimally invasive thoracic surgery, increased day-case slots for therapy, equal opportunities for patient access to clinical trials, a boost to tissue biobanks, and easing of drug reimbursement. There is room for improvement across all countries and all domains. The aim is to ensure that there are fast-track referral pathways, effective psychological support services, strong public health regulations and access to high-quality treatment. There is also a need for continued development of a more robust understanding of LC epidemiology and risk factors, more extensive, earlier and co-ordinated use of molecular testing biomarkers achieving rapid turnaround times and encouragement for developing LB [60]. Comprehensive and up-to-date national cancer control plans can be used to guide these improvements and to better evaluate implementation. The impact is not immediately visible after policy implementation and a time lag has to be taken into account. LC guidelines should encompass clear referral pathways, timeframes and quality indicators.

#### 3.2.2. Colorectal Cancer

Colorectal cancer (CRC) is the third most common malignancy in Europe, posing a severe health problem. In 2020 around 1.9 million incidence cases and 0.9 million deaths happened worldwide [61]. In 2015, the economic burden of CRC across Europe was EUR 19 billion [62,63]. The mean cost for managing a patient with CRC varies widely, and countries with similar gross domestic product per capita have widely varying healthcare costs. The incidence of CRC is generally higher for men, and the risk of the disease increases with age. Risk factors include consumption of processed meats, alcoholic beverages, smoking and obesity [64,65,66,67]. The last decade has seen innovations in clinical practice in diagnosis and treatment, even for advanced diseases. However, advances are unevenly available, and five-year survival still ranges from 49% to 68% across the member states [65]. Screening can substantially reduce the risk of death through the detection and removal of precancerous lesions. Options include stool-based blood tests, faecal immunochemical tests, endoscopic methods and colonoscopy, with CT colonography and analysis of biomarkers in the stool, blood, or breath under development [68,69]. A 2003 EU Council recommendation included national CRC screening, and a 2007 declaration re-iterated the need for more outstanding official support for CRC screening and standardisation of practices across Europe [70,71]. Organised screening programs exist in Finland, France, Slovenia, the Netherlands and the United Kingdom. Nevertheless, there have been no European CRC screening recommendations from the scientific community since 1999, and only Finland, France, Germany, Italy and UK have screening guidelines. Participation rates in screening vary among countries and settings. However, they have typically been below 40%, influenced by insurance status, access to primary care, costs, logistic challenges, lack of provider involvement, language barriers, cultural beliefs and lack of awareness [67,69]. Medical treatment is not standardised globally, but in Europe, European Society for Medical Oncology (ESMO) guidelines cover early colon, rectal, metastatic and familial risk of CRC [72]. Endoscopy demand outstrips supply in many countries with the risk of longer waiting times for higher-risk symptomatic patients. Only the Netherlands and the UK prioritise these patients [70,73]. Targeted biological treatments are now contributing to more prolonged survival, and most European countries have added these treatments to their reimbursement schemes. However, in the UK, they are severely limited and not reimbursed as standard [70]. Nationwide, repeated resectability online assessment by centralised multidisciplinary teams offered to all Finnish hospitals and leading to resections at high-volume centres resulted in improved conversion and resection rates, with impressive survival [74]. Testing of tumor material for Microsatellite Instability (MSI) in de novo diagnosed tumors is not widespread to date, yet may offer selected MSI high patients the chance of receiving neoadjuvant immunotherapy with promising results [75]. Real-time monitoring of ctDNA can provide insights into tumour evolution, and there is an urgent need to accelerate its integration into routine CRC care [76]. Certain observational studies have shown that when ctDNA is detected after curative-intent therapy without further adjuvant treatment, a very high risk of recurrence (>80%) is occurring. A recent study, published in 2022, showed that a ctDNA-guided approach reduced adjuvant chemotherapy use without compromising recurrence-free survival. Still, more data are needed to rule out the possibility that the treatment of ctDNA-positive patients with chemotherapy may have delayed rather than prevented recurrence in some instances [77].

##### National Variations in CRC Experience

In Germany, CRC is identified each year in more than 37,000 men and 36,000 women, mainly in those around 70 years of age and above. Cancer registries and their data need improvement. Colonoscopy is largely reimbursed, and increased use has coincided with a decline in CRC incidence and mortality over the last decade. However, screening is opportunistic, and some confusion over reimbursement and concern over its costs is associated with low take-up of screening. Coordination between ambulatory and secondary care is poor, impeding best care and causing unnecessary delays in treatment. Guidelines from 2008 exist for diagnosis, treatment, and post-treatment surveillance. Surgery is the most common treatment, increasingly supplemented by pharmaceutical treatment [18,68].

In Italy, the cost of care varies according to the stage in the care pathway. The Covid pandemic led to the suspension of screening programs, threatening significantly higher numbers of more advanced cases and even increased mortality [78]. 

In Croatia, CRC is one of the most common cancer types (17% among males and around 8% in females) and represents essential health and economic burden, with drug costs accounting for nearly half of total expenditure [79].

In 11 Balkan countries (Croatia, Bosnia, Albania, Bosnia and Herzegovina, Bulgaria, Croatia, Greece, Montenegro, Romania, Serbia, Slovenia, The Republic of North Macedonia, and Turkey), Turkey, Romania, and Greece had the highest number of new cases but predominated among all cancer cases in Slovenia, Croatia, and Romania. Compared with other cancer sites, the highest percentage of deaths was in Croatia, Bulgaria, and Romania. Montenegro had the best results. GDP per capita level showed a strong correlation with CRC indicators. Limited access to screening was linked to a seemingly lower incidence of CRC in lower socio-economic populations but with a subsequent increase in mortality. Other studies show that Slovenia, Croatia, Serbia, Bulgaria, and Montenegro, as well as the other Central and Eastern European countries, have a higher mortality-to-incidence ratio in comparison to Western European and Nordic countries—which have higher health expenditure per capita. Economic factors can influence the epidemiology of CRC, and the heavy CRC burden in the Balkan region may be one of the indexes of economic development. More cost-effective national reimbursement policies could also provide actual savings to the Balkan health systems [80].

In the United Kingdom, CRC accounts for an increasing health burden. National screening invites older populations to biennially partake in guaiac faecal occult blood testing (FOBT). The adequacy of UK endoscopy services has been questioned, and waiting times for colonoscopy continue to vary widely from a few weeks to over 6 months. Surgery is a primary mode of treatment for CRC patients. Current guidelines from the National Institute for Health and Care Excellence (NICE) and medical societies include details on patient-centred care, treatment access, multidisciplinary teams, diagnosis, surgery, radiotherapy, chemotherapy, surveillance, recurrence, and palliative care. Provision is suitable for data collection, awareness, choice of treatment and endoscopy centres and national cancer plans are good, but NICE should work faster, guidelines should be more precise and more recent, and regional variations in care should be overcome [71,81].

Sweden has no national screening recommendation, and screening coverage is low. Surgical procedures, including colonoscopies, are recorded, although incompletely [82,83]. There are national treatment guidelines for CRC, which are regularly updated [84]. The national registries in CRC are very extensive and comprehensive, and treatment results are thus followed extensively [85].

Finland has a national screening program and screening coverage is among the highest in Europe. The national treatment guidelines are regularly updated [86,87].

##### CRC Needs

Need for more (and more precise) guidelines on screening and treatment and improvements to endoscopy services. Inequalities of access to care, high-quality treatment, and inequalities across regions and states must be tackled as a priority. Guidelines on screening and treatment need updating and following to account for advanced techniques such as real-time monitoring of ctDNA. Participation rates in screening need to be improved.

#### 3.2.3. Breast Cancer

Breast cancer (BC) is by far the most frequently diagnosed cancer and the leading cause of death in women across Europe (42,000 a year) and presents a significant public health problem and substantial economic burden [34,88]. Improvements in management and treatment, early diagnosis and systematic screening have significantly reduced mortality in the EU, but survival rates following treatment vary by 20% between countries. Although BC is treatable and potentially curable, up to 10% of patients are diagnosed at an advanced stage in developed countries. Even if diagnosed early and appropriately managed, 20–30% of patients experience progression to metastatic BC [88,89]. European Commission guidelines for organised mammography screening programmes in asymptomatic women have been regularly updated and expanded. Screening is available in all European countries, but not all eligible women get screened—ranging from 6% to 90% [10,11,90]. BC is treated by surgery, radiotherapy, chemotherapy and endocrine therapy, which, together with the targeted therapies developed in recent decades, have greatly improved overall survival. Immunotherapies may be added to the therapeutic arsenal against BC in the near future [91]. There is growing evidence of the prognostic merits of LB in primary BC and of the clinical utility of CTCs in assigning people with metastatic BC to either chemotherapy or hormonal therapy [76,92]. The high treatment costs are due to the length of survival achieved nowadays with combination endocrine therapy in the largest subset of BC, hormone-receptor-positive women, resulting in long periods on medication as well as individual per patient costs of novel therapies in HER2+ and triple-negative BC patients [93,94]. For metastatic BC treatment, a pan-European approach is needed along with multistakeholder collaboration to overcome challenges in obtaining optimal cancer care, particularly for those with poorer healthcare, including improvements in healthcare personnel education, policy-related issues, support for patients, caregivers and employers, investment in medical innovations and efficient use of healthcare resources, along with the implementation of high-quality mBC treatment guidelines in all countries. Dialogue between patients, oncologists, PCPs and other HCPs is needed for enhanced understanding and shared treatment decision making [95]. 

##### National Variations in BC Experience

In the United Kingdom, patient advocacy has turned BC into a political priority, with government support for initiatives to encourage early diagnosis, including for younger and older women. However, uptake of screening programmes varies across demographic groups. The costs related to the primary treatment pathway are covered, from diagnosis through treatment. However, compliance and adherence to guidelines vary across regions, leading to differences in the diagnosis, treatment and care. Detection rates at stage I or II range widely—from 36.3% to 88.0%, and regional differences in numbers of skilled radiographers and radiologists lead to varying wait times between diagnosis and treatment. Innovative funding mechanisms are required to promptly improve access to efficacious anticancer medications at a sustainable cost [26,96]. 

In Italy, all breast care is free at the point of care, including screening and treatment for early BC. BC is a political priority, driven partly by patient advocacy groups. Nevertheless, uptake varies, with 94% in northern and 40% in southern Italy—figures reflected in mortality and incidence rates. Differences in quality of care are exacerbated by limited health literacy and awareness. Better organisation of care is needed to use existing technologies better and manage access to new technologies such as digital mammography tools. Clinical guidelines at international, national and regional levels are not systematically followed. However, it is easier for hospitals to get reimbursement for procedures and pharmaceuticals when they conform to national guidelines. Healthcare infrastructure needs updating to ensure timely treatment following clinical guidelines and reduce wait times during treatment [26,97]. 

In Spain, BC is the primary malignant neoplasm among women. However, the mortality rate is the lowest in Europe (23.4 per 100,000 inhabitants in 2018) due to a national strategy—also driven by patient advocacy—promoting quality in cancer care, implementing early detection programmes, and advances in diagnosis and treatment. Benefits cover up to 18 months of unemployment. Nevertheless, there is regional variation in the implementation of the national screening strategy for early detection and treatment availability because drug budgets are regional—the main challenges to policymaking and accessing treatment for early BC and a brake on uptake of innovation. There are no national guidelines for treating early BC, but medical society guidelines exist [26,98]. 

BC is the most frequent cancer among French women and the leading cause of cancer-related death. National cancer plans have organised national screening (breast and colon cancer and recently cervical cancer), promoting prevention, rein-forcing care pathways, and improving life during and post-cancer [91].

BC poses a substantial economic and social burden in Central and Eastern Europe, where survival rates are still lower than in Western Europe. Access to new medicines varies widely, and shortages affect even medicines with well-established use, including in the list of essential medicines. Usage of breast irradiation is limited and differs from country to country. Only a few countries in the region have organised early detection; PET/CT is not universally available. Few countries use MRI routinely for diagnosis, with core needle biopsy most widely used. National guidelines are widely followed, but there are vast differences in national healthcare services and their financing. Genetic testing is not widely covered by public expenditures [99].

##### BC Needs

It is urgent to equalise the strikingly different survival rates following treatment between countries. It is also urgent to improve the current rate of late diagnosis, which is itself partly the result of patchy participation in screening. Professional expertise needs to increase in availability and skill levels, particularly in radiology. The development of LB tools in the earlier stage of BC is crucial [100]. Optimal treatment is still impeded by inadequate reimbursement of new therapies and diagnostics. Developing patient advocacy in many countries could improve BC’s political attention level and resource allocation.

#### 3.2.4. Melanoma

Melanoma (MM) is less common than basal cell and squamous cell skin carcinoma but is far more aggressive and accounts for 90% of the deaths associated with cutaneous tumours [101]. Incidence is rising worldwide, causing around 55,500 deaths annually [102]. Incidence is high in Northern and Western Europe and apparently lower in the Southern and Eastern European countries—perhaps due to skin type but also to under-diagnosis and poor registration. Men are more often diagnosed later with more advanced tumours and worse outcomes, correlated to more advanced stages of disease at diagnosis [103]. Diagnostics are via clinical examination of the suspected tumour, physical examination for signs of lymph node disease, and detailed examination of the lesion using digital or analogic dermatoscopes, total body photography and reflectance or confocal microscopy. Sentinel lymph node dissection is routinely offered in the case of larger tumours as a staging procedure, and there is clear evidence of independent prognostic value, but it has no therapeutic benefit. Therefore, it is justified for staging of patients and guidance for follow-up and adjuvant treatment. Targeted therapy and immunotherapy are nowadays offered as an adjuvant as well as in metastatic settings to patients to patients with stage III melanoma, and recently immunotherapy has been approved in Europe in stage II [101,104,105,106]. Surgical therapy or systemic treatment (including targeted therapy and immunotherapy) is offered for distant metastasis. Novel therapeutic approaches now appearing are improving patients’ overall survival and quality of life and making early access to treatment—and accurate targeting—all the more important [104,107]. An international survey across 30 European countries concluded that 27% of 19,600 patients with advanced MM did not get access to the standard first-line therapy, often because of long delays between marketing authorisation and drug reimbursement [22,108]. Guidelines from European medical organisations promote the integration of care between medical and paramedical specialities and better management of MM from primary melanoma diagnosis through advanced disease palliation. European consensus on the management of MM is still incomplete. Early diagnosis of MM ensures a simple treatment with survival near 100% of patients. Most cutaneous melanomas can be detected at early stages by clinical examination. However, early diagnosis of tumours is still far from optimal due to multiple factors, including lack of education of patients, proper examination of patients with total body examination and limited access to dermatologists. Earlier diagnosis could be improved by algorithm-assisted diagnosis to improve accuracy at first presentation, and publicly accessible technology for self-diagnosis, given the uneven degree of awareness at population levels [101,109]. Mobile phone-based app technology is being evaluated as a support tool for self-screening/diagnosis (SkinVision|Skin Cancer Melanoma Detection App|Skin-Vision and others) [110]. 

##### National Variations in MM Experience

In France, access to therapeutic innovations is mainly through participation and inclusion in clinical trials, local hospitals, or through the French national early access program, after the national health system-imposed cutbacks, such as stopping funding ipilimumab in advanced MM. Equity of access to new anticancer drugs is consequently not guaranteed. Hospital pharmacies have formed consortia to negotiate prices with pharmaceutical companies directly [22,111]. 

In Denmark, the incidence of MM has increased during the past decades [112]; however, the survival of patients with metastatic disease has improved significantly after the introduction of anti-PD-1 drugs [113]. Since 2018, novel therapies in the metastatic (including the combination of an-ti-CTLA-4 plus anti-PD-1) and adjuvant settings have been reimbursed.

In Spain, similarly to France and other countries, access to therapeutic innovations is mainly through participation and inclusion in clinical trials available only in referral hospitals. Access of patients to public health primary care is universal and fast-track consultation to dermatologists integrated teledermatology in most regions. The national health system approves reimbursement of therapies in MM with years of delay after the approval of the indication by the EMA and imposed cutbacks, such as stopping funding for adjuvant therapies in stages IIIA and IIIB [105]. Equity of access to new anticancer drugs is consequently not guaranteed compared to other European countries.

Germany is seeing an increased incidence of cutaneous melanoma (CMM), probably due to the introduction of a population-wide and reimbursed skin cancer screening. Prevention campaigns have failed to reverse the trend, particularly in older age groups. Treatment is reimbursed without restriction if a potentially life-threatening condition cannot be adequately treated by appropriately authorised medicinal products [101,114]. 

In Italy, incidence increased for both sexes in all age groups, making it the third most frequent malignancy in both sexes under the age of 50. Based on national guidelines for the management of CMM, a diagnostic and therapeutic patient care pathway has been adopted in some regions to reduce inequalities and variability in patient management. Novel therapies are reimbursed [115,116].

In Belgium, about 2800 melanomas are diagnosed, and 300 people die yearly. Novel therapies are considered a cost-effective option in the first-line treatment of advanced MM [117]. 

In Croatia, MM is usually diagnosed in advanced stages, despite public awareness campaigns, and represents a significant public health problem with a high burden and costs. Incidence rates are rapidly increasing in Mediterranean countries, but screening for melanoma is not yet common. Standard treatment for metastatic melanoma shows only modest efficacy and unquantified survival benefit. Nearly half the costs come from drugs and hospitalisation, with only a tenth for dermatoscopy. The goal should be to redirect the savings towards programmes for the prevention and early detection of MM or invest in drugs to treat advanced-stage patients. Unlike in some western countries, nationwide melanoma screening is still not obligatory [104,118].

In Poland, approximately 1400 patients die annually from skin MM. Public financing of new drug therapy in Poland is usually performed within a separate financial path authorised explicitly by the minister for health and with strict requirements, including appropriate values of laboratory parameters and physical condition, forcing the selection of patients [119].

##### MM Needs

European consensus on the management of melanoma would raise standards of timely diagnosis and innovative treatment—although that also requires improvement in reimbursement of testing and therapies [101,120,121]. 

## 4. Discussion

Policy developments should reflect the evident need to drive access, diagnosis, innovation, investment and research forward and redress the inequalities across Europe and among different populations and pathologies [122]. An overall cancer plan—desirable in itself—is not sufficient, as a cancer plan is needed for each type of cancer. Nevertheless, at a general level, it is evident across the board that cancer registries are currently inadequate, non-standardised and lacking essential clinical data. There are vast differences in incidence and survival of many cancers from state to state, and these differences are also reflected in screening and access to appropriate therapies and expertise. These regional variations are not cultural but the result of policy and political will to look beyond cost-benefit calculations. All countries should have the same objective of equal access to optimal care and equal outcomes. Moves are needed to systematically speed the process between early symptoms and diagnosis and between diagnosis and treatment. Uniform reporting standards, more comprehensive implementation of multidisciplinary teams and more excellent centralisation of specialised care in high-volume centres would assist. Measures are needed to overcome the lack of accurate data on costs, treatment modalities, outcomes—and the associated direct and indirect costs. The challenges in data sharing—both because of General Data Protection Regulation (GDPR) and the still-widespread tendency among hospitals to guard their own data—need to be overcome, both in the interests of advancing research and best practice and in the interests of patients’ rights to know where the best results are being achieved. There is also a general need for increased awareness of cancer risks and prevention across all populations.

Furthermore, what can EBCP provide? The actions envisaged by the Commission in its communication to the European Parliament and the Council offer many evident benefits for cancer in general and LC, CRC, BC and MM. They include:

Better diagnosis—with help from the Knowledge Centre on Cancer and the European Cancer Imaging Initiative, and the proposed EU Cancer Screening Scheme, as well as through the revision and extension of the Council Recommendation on cancer screening and new guidelines and quality assurance schemes on screening and diagnosis.

Saving lives—by better diagnostics and treatment and improved access, as well as better follow-up (notably for colorectal and cervical cancer, and updating the existing guidelines on BC, including accreditation and certification programmes); more comprehensive vaccination (and related infrastructures) to pursue the elimination of cancers caused by Human papillomavirus.

Better innovation—through improved access to the benefits of research and digitization, Horizon Europe partnerships, a dedicated cancer research ecosystem, wider data sharing (notably with the European Health Data Space), and repositories of digital twins.

Better product development—through the Partnership on Personalized Medicine that will identify priorities for research and education in personalised medicine, support research projects relevant to cancer prevention, diagnosis and treatment, promoting well-powered clinical studies on cancer care.

More comprehensive access—through mainstreaming equality actions ranging from screening and care to infrastructure, focused on redressing differences and gaps across regions and member states [1,3]. 

## 5. Conclusions

There is good reason for some optimism about the future of cancer care in Europe with the adoption of EBCP and the Cancer Mission. However, as with all EU policy, the announcement of intentions—even as constructive as in the EBCP—is not the same thing as delivery on the promise. Policy momentum needs to be maintained now more than ever, and organised pressure from stakeholders has an essential role in that process. In other words, if cancer stakeholders—patients, carers, HCPs, authorities, etc.—wish to see EBCP lead to improvements, they must maintain contact with policymakers to ensure that intentions and undertakings are transformed into results.

## Data Availability

Not applicable.
